# *In situ* TEM observation of liquid-state Sn nanoparticles vanishing in a SiO_2_ structure: a potential synthetic tool for controllable morphology evolution from core–shell to yolk–shell and hollow structures†

**DOI:** 10.1039/c9na00782b

**Published:** 2020-02-03

**Authors:** Shilei Zhu, Mai Thanh Nguyen, Tomoharu Tokunaga, Cheng-Yen Wen, Tetsu Yonezawa

**Affiliations:** Division of Materials Science and Engineering, Faculty of Engineering, Hokkaido University Kita 13 Nishi 8, Kita-ku Sapporo Hokkaido 060-8628 Japan tetsu@eng.hokudai.ac.jp mai_nt@eng.hokudai.ac.jp; Department of Materials Design Innovation Engineering, Faculty of Engineering, Nagoya University Furo-cho, Chikusa-ku Nagoya 464-8601 Japan; Department of Materials Science and Engineering, National Taiwan University No. 1, Section 4, Roosevelt Rd, Da'an District Taipei City 10617 Taiwan; Institute for the Promotion of Business-Regional Collaboration, Hokkaido University Kita 21 Nishi 11, Kita-ku Sapporo Hokkaido 001-0021 Japan

## Abstract

Precise design of hollow nanostructures can be realized *via* various approaches developed in the last two decades, endowing nanomaterials with unique structures and outstanding performances, showing their usefulness in a broad range of fields. Herein, we demonstrate the formation of SnO_2_@SiO_2_ hollow nanostructures, for the first time, by interaction between liquid state Sn cores and SiO_2_ shell structures inside Sn@SiO_2_ core–shell nanoparticles with real-time observation *via in situ* transmission electron microscopy (TEM). Based on the *in situ* results, designed transformation of the nanoparticle structure from core–shell Sn@SiO_2_ to yolk–shell Sn@SiO_2_ and hollow SnO_2_@SiO_2_ is demonstrated, showing the controllable structure of core–shell Sn@SiO_2_ nanoparticles *via* fixing liquid-state Sn inside a SiO_2_ shell which has a certain Sn containing capacity. The present approach expands the toolbox for the design and preparation of yolk–shell and hollow nanostructures, thus providing us with a new strategy for fabrication of more complicated nanostructures.

## Abstract 2

Controllable nanostructure evolution of Sn@SiO_2_ from core–shell to yolk–shell and hollow has been achieved by the interaction between liquid-state Sn and SiO_2_ shell, based on our *in situ* TEM observation results.

## Graphical abstract

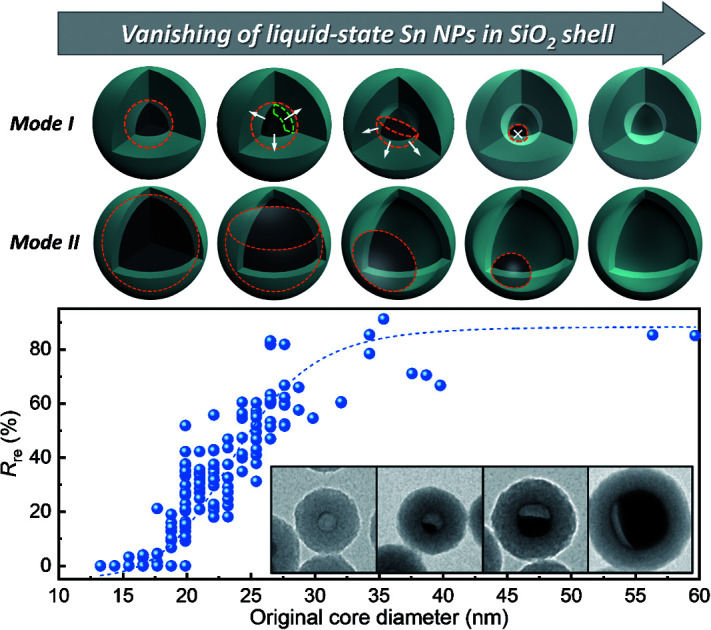

## Introduction

Hollow nanoparticles have been applied in a broad range of applications, such as catalysts,^1^ energy conversion and storage,^2^ sensors,^3^ and biomedicines,^4^ due to their unique properties including large specific surface area, low density, *etc.*, determined by their ingenious structures. Much work so far has focused on the synthesis of hollow nanoparticles, which basically can be classified into three strategies: (1) a hard templating pathway, (2) a soft templating pathway, and (3) a self-templating synthetic route which includes surface-protected etching, Ostwald ripening, the Kirkendall effect, and galvanic replacement.^5–10^ Generally, all these strategies consist of two basic steps, including (1) formation of a precursor for the hollow structure (*e.g.*, core–shell nanostructures) and (2) creation of a hollow space by various methods including etching of cores, removal of templates, net outward diffusion, *etc*.^11^

So far, the formation of a hollow structure in most of the research has been carried out and observed *via ex situ* techniques, in which the transient information involved during the hollowing process was missing. To gain more insight, *in situ* transmission electron microscopy (TEM) has been used in recent research to observe the hollowing process caused by the galvanic reaction^12,13^ and the Kirkendall effect.^14,15^ E. Sutter *et al.* used liquid-cell electron microscopy to investigate galvanic replacement reactions between Ag nanoparticle templates and aqueous Pd salt solutions. They observed the transformation from Ag nanoparticles into hollow Ag–Pd nanostructures.^12^ Chee *et al.* used liquid-cell electron microscopy for observation of the hollow Ag nanostructure with void formation due to the nanoscale Kirkendall effect occurring in conjunction with galvanic replacement.^15^ Several studies have reported the *in situ* observation of hollow structure formation by removing core templates from the core–shell structures. Yang *et al.* observed that when exposed to electron beam irradiation, ZnO@Al_2_O_3_ core–shell nanowires can be transformed into ZnO@Al_2_O_3_ composite nanotubes *via* local etching of ZnO cores.^16^ Zhu *et al.* observed the transformation of Se–C core–shell nanoparticles into yolk–shell nanoparticles and hollow nanoparticles by electron beam irradiation, which induced the evaporation of embedded Se.^17^

In this study, we report the formation of SnO_2_@SiO_2_ hollow nanostructures, for the first time, by interaction between liquid state Sn cores and SiO_2_ structures that were synthesized through wet chemistry inside Sn@SiO_2_ core–shell nanoparticles, with real-time observation *via in situ* TEM. According to the *in situ* observation, diffusion of liquid state Sn at 300 °C contributes to the moving-out of embedded Sn cores, creating a cavity inside SiO_2_ shell structures. Based on this understanding, to the best of our knowledge, we demonstrate the synthesis of yolk–shell Sn@SiO_2_ and hollow SnO_2_@SiO_2_ nanoparticles from core–shell Sn@SiO_2_ nanoparticles for the first time, showing nanostructure control *via* interaction between liquid state metal and the SiO_2_ shell structure. Based on these results, this new strategy is capable of being applied in the design and preparation of yolk–shell, hollow nanostructures or more complicated nanostructures with the heating method which is expected to be achieved by using lasers or microwaves.

## Results and discussion

### Structural characterization

**Fig. 1:**
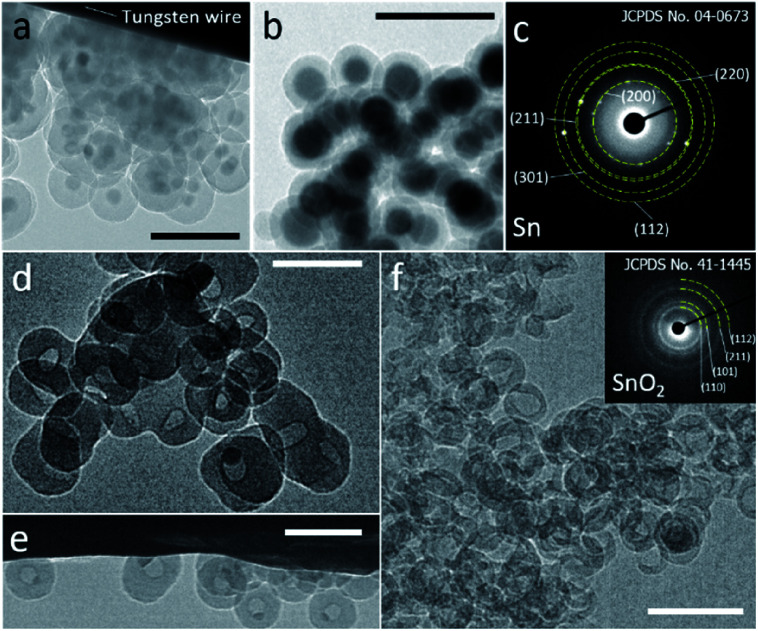
(a and b) TEM images of Sn@SiO_2_ samples with two different Sn core sizes ((a): 20 nm and (b): 30 nm) and shell thicknesses ((a): 20 nm and (b): 8 nm), which are loaded on a tungsten wire inside a TEM heating holder observed in a vacuum at room temperature (25 °C). (c) The SAED pattern of Sn@SiO_2_ shown in (a). TEM images of Sn@SiO_2_ after heating from room temperature (25 °C) to 300 °C in a vacuum performed in a high-voltage TEM operated at 1000 kV under (d and e) strong electron beam conditions (37.3 pA cm^−2^) and (f) weak beam conditions (2.7 pA cm^−2^); inset: the SAED pattern showing the as-formed SnO_2_. All scale bars are 100 nm.

A modified reverse-emulsion method was used for uniform and controllable SiO_2_ encapsulation of Sn nanoparticles which were prepared though a well developed hot-injection method.^18^ Sn@SiO_2_ core–shell nanoparticle samples with two dimensions were produced: (a) a 20 nm core with a 20 nm shell (Fig. S1, see the ESI†) and (b) a 30 nm core with an 8 nm shell (Fig. S2, see the ESI†). The samples were loaded on a tungsten wire (as shown in Fig. 1a and b, respectively, in a vacuum at 25 °C) and heated inside a TEM heating holder for *in situ* TEM observation (Fig. S3, see the ESI†). Several Sn@SiO_2_ core–shell nanoparticles in Fig. 1a were examined by selected area electron diffraction (SAED). The diffraction spots in the corresponding pattern shown in Fig. 1c are indexed to tetragonal Sn (JCPDS no. 04-0673), indicating the existence of metallic Sn cores. After completing the whole heating process (from 25 °C to 300 °C in a vacuum) in a TEM and cooling down to 25 °C, most of the Sn cores disappeared and some decreased in size, forming hollow and yolk–shell nanostructures as shown in Fig. 1d–f. Sn@SiO_2_ nanoparticles shown in Fig. 1a were observed under strong electron beam conditions (37.3 pA cm^−2^). Under these conditions, significant deformation of SiO_2_ shells occurred with interconnections between them (Fig. 1d). Meanwhile, according to the SAED pattern of the hollow and yolk–shell nanostructures in Fig. 1d (Fig. S4, see the ESI†), the rings are indexed to SnO_2_ (JCPDS no. 41-1445) while several dim spots are indexed to the (200) and (101) planes of tetragonal Sn (JCPDS no. 04-0673), demonstrating that the transformation of Sn to SnO_2_ occurred during the heating process, probably due to the interaction with the dangling bonds in the porous SiO_2_. In order to avoid the effect of beam irradiation, weak electron beam conditions (2.7 pA cm^−2^) were applied when observing Sn@SiO_2_ nanoparticles as shown in Fig. 1b. After heating, most of the Sn cores disappeared, leaving behind a hollow SiO_2_ structure without significant deformation (Fig. 1f), and its corresponding SAED pattern (the inset image in Fig. 1f) is indexed to SnO_2_ (JCPDS no. 41-1445), indicating the existence of SnO_2_.

### Dynamic motion of Sn nanodroplets inside the SiO_2_ structure

**Fig. 2:**
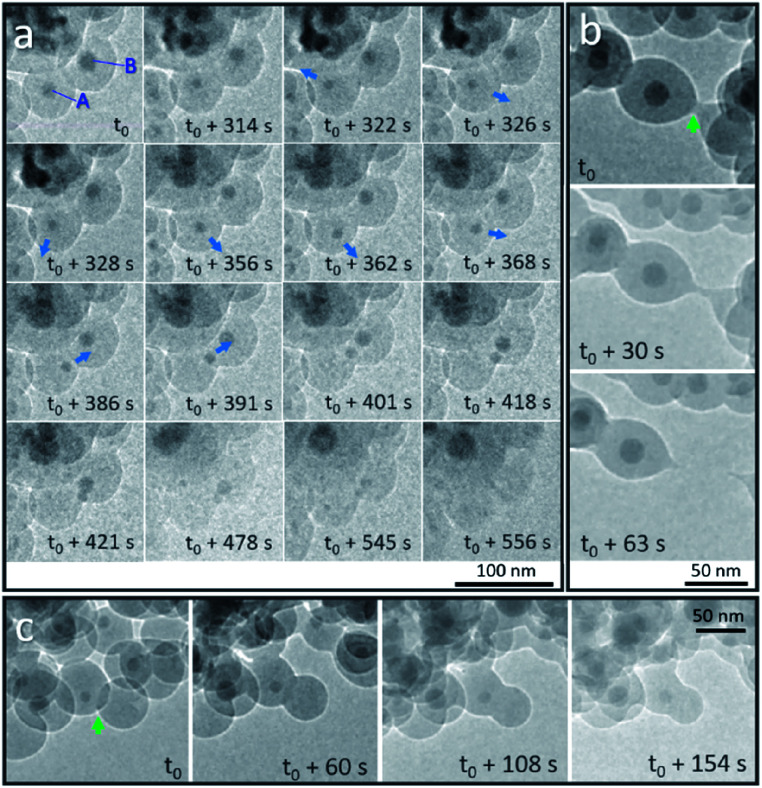
*In situ* observation of Sn@SiO_2_ at 300 °C in a vacuum under strong electron beam conditions (37.3 pA cm^−2^). (a) Random motion and a gradual decrease in the size of Sn droplets that are embedded inside the SiO_2_ shell indicate the possible diffusion of Sn at an elevated temperature (300 °C). The blue arrows show the movement direction of Sn droplets according to the movie (ESI Movie 1, see the ESI†). Deformation of the SiO_2_ shell structure occurred during observation: (b) breakage of the connected SiO_2_ shell of two Sn@SiO_2_ core–shell particles and (c) connection of two individual particles are shown (marked with green arrows).

We first discuss the dynamic motion of Sn nanodroplets inside the deformed SiO_2_ structure at an elevated temperature under electron beam irradiation according to the observation of detailed structural evolution and transformation that was recorded *in situ* during the heating process. When the temperature exceeds the melting point (*T*_m_) of metallic Sn (bulk: 231 °C, 20 nm in diameter: 223 °C),^19^ the SiO_2_ encapsulated solid-state Sn cores are transformed to a liquid state as Sn nanodroplets. Fig. 2a shows the dynamic motion of Sn nanodroplets inside the SiO_2_ structure at 300 °C in a vacuum under electron beam irradiation (37.3 pA cm^−2^) captured from the movie (ESI Movie 1, see the ESI†). In the initial state, Sn cores A and B, with initial diameters of 16.9 and 21.2 nm, respectively, were located at the central positions of two adjacent Sn@SiO_2_ core–shell nanoparticles with clear boundaries in between (Fig. 2a, *t* = *t*_0_). As the heating proceeded, both core A and B decreased in size (A: 11.3 nm and B: 16.6 nm, Fig. 2a, *t* = *t*_0_ + 314 s) and core A started to move away from the central position (Fig. 2a, *t* = *t*_0_ + 322 s). By *t*_0_ + 362 s, dynamic motion in random directions inside the SiO_2_ shell is observed. The instant movement directions of Sn cores are shown with blue arrows marked in the TEM images according to the movie. After *t*_0_ + 368 s, core A started to move along a fixed direction toward core B and reached the edge of the SiO_2_ shell at *t*_0_ + 391 s. Meanwhile, core B moved in a similar manner toward the outer edge of the SiO_2_ shell and met core A at *t*_0_ + 421 s. During the heating process, both core A and B gradually became invisible by contrast in the SiO_2_ structure (Fig. 2a, *t* = *t*_0_ + 556 s). The observed movement and disappearance of Sn cores may provide evidence for the vanishing of metal Sn inside the SiO_2_ structure. The dynamic motion of Sn cores was probably caused by the dynamic wetting conditions which are resulted from the variation of the environment around Sn cores, such as the interface curve and pressure. It is worth mentioning that no clear cavity could be observed after Sn cores left their previous central positions during heating, which probably can be explained by the structural deformation and viscous flow of SiO_2_ under high-dose electron beam irradiation.^20–23^ This structural deformation of the SiO_2_ shell is clearly observed in Fig. 2b and c during heating (300 °C) under electron beam irradiation (37.3 pA cm^−2^). Fig. 2b shows the breakage of two connected Sn@SiO_2_ core–shell nanoparticles. The connection area existed initially between these two Sn@SiO_2_ core–shell nanoparticles (Fig. 2b, *t* = *t*_0_, marked by a green arrow), and then it stretched out in length (Fig. 2b, *t* = *t*_0_ + 30 s), and finally broke accompanied by the formation of a tail-like morphology (Fig. 2b, *t* = *t*_0_ + 63 s) (ESI Movie 2, see the ESI†). Fig. 2c exhibits the merging and connection of two individual particles. The green arrow in Fig. 2c shows the overlap and clear boundaries between SiO_2_ shells by contrast (Fig. 2c, *t* = *t*_0_). As heating progressed, these boundaries became blurred (Fig. 2c, *t* = *t*_0_ + 60 s) and then disappeared (Fig. 2c, *t* = *t*_0_ + 108 s), indicating connection between the SiO_2_ shells and merging of the two individual particles (ESI Movie 2, see the ESI†). It is reasonable to believe that the deformation and viscous flow were not induced by simply heating, since in our case, the heating temperature 300 °C is far below the ∼0.7 *T*_g_ (glass-transition temperature) of SiO_2_ (*T*_g_ = 1373 K) and cannot cause viscous flow of SiO_2_ for a significant deformation rate.^23^ In addition, the temperature increase caused by energy transfer from the electron beam under our conditions is also far below 0.7 *T*_g_ according to the calculation given by Zheng *et al.*^20^ In order to minimize the effect from electron beam irradiation, Sn@SiO_2_ core–shell nanoparticles distributed in other places, which were far from the focus area for movie recording during the heating process, were also observed after cooling down to 25 °C. Reduced deformation is observed compared to the SiO_2_ structures in the focus area, whereas the hollow nanoparticles still formed (Fig. 1e), which indicated that electron irradiation is not necessary for the formation of hollow structures in our case.

### Cavity formation induced by vanishing of liquid-state Sn cores

**Fig. 3:**
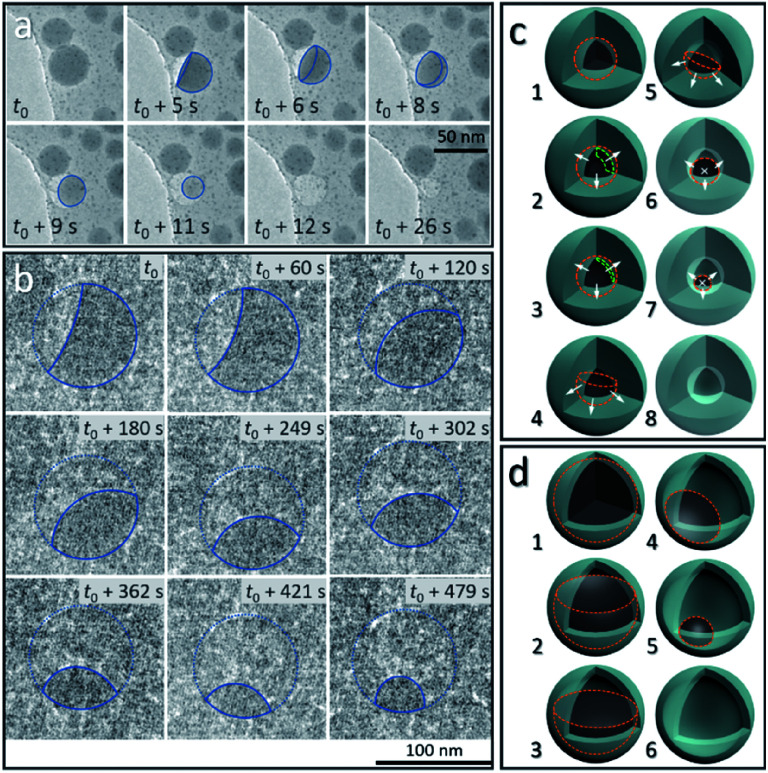
*In situ* observation of the disappearance of a single liquid-state Sn core under (a) strong electron beam conditions (37.3 pA cm^−2^) (mode I: a balloon-like liquid-state Sn layer forms and decreases in thickness due the outward diffusion of Sn into the outside SiO_2_ structure and disappears in seconds) and (b) weak electron beam conditions (2.7 pA cm^−2^) (mode II: the remaining Sn core slowly decreases in size due to the diffusion of Sn and gradually disappears) at 300 °C in a vacuum, respectively. The blue solid lines in (a and b) show the outline of the remaining Sn core during the diffusion process. The position of the SiO_2_ shell in (b) is outlined using a blue dashed line. Schematics of the two different diffusion modes: (c) mode I and (d) mode II, illustrating the diffusion process shown in (a and b). In (c and d), the outline of the remaining Sn core and the thickness of the liquid Sn layer are marked using orange dashed lines and green dashed lines, respectively. The white arrows are used for showing diffusion directions (the white crosses “×” represent the arrow directed into the page).

From the above discussion, the observed dynamic movements and vanishing of Sn cores show the possible occurrence of Sn diffusion in the SiO_2_ structure. Fig. 3 shows the *in situ* observation of two different pathways, mode I and mode II, for the disappearance of Sn cores embedded in SiO_2_, providing a visual demonstration of how the cavity forms inside the silica structure induced by increasing the temperature in a vacuum.

Fig. 3a shows the vanishing process in mode I of Sn cores inside the SiO_2_ structure at 300 °C in a vacuum, captured from the movie (ESI Movie 3, see the ESI†). This process is illustrated in Fig. 3c (8 stages marked by numbers 1–8). The Sn core with a size of 38.5 nm was embedded in the deformed continuous SiO_2_ structure which was derived from the interconnected SiO_2_ shell, induced by electron beam irradiation (37.3 pA cm^−2^) as discussed above. At 300 °C, this Sn core first melted to a liquid droplet (stage 1 in Fig. 3c, outlined with an orange dashed line) and after heating, showed lower contrast compared with the as-formed ultra-small particles (∼2 nm) distributed around it, indicating that the Sn core did not exist as a filled spherical droplet but a balloon-like spherical liquid layer that adhered on the inner surface of the cavity in the SiO_2_ structure (Fig. 3a, *t* = *t*_0_) (stage 2 and 3 in Fig. 3c; the layer is shown by using a green dashed line). It is reasonable to consider that uniform diffusion of Sn may occur throughout the outward directions perpendicular to the Sn–SiO_2_ interface (shown as white arrows in stage 2 and 3 in Fig. 3c). As the diffusion proceeded, the liquid Sn layer decreased in thickness (stage 3 in Fig. 3c) and then broke with the appearance of a hole (Fig. 3a, *t* = *t*_0_ + 5 s) (stage 4 in Fig. 3c). The balloon-like spherical liquid layer turned to a bowl-like structure and then vanished quickly with the decrease in the area of the Sn–SiO_2_ interface (Fig. 3a, *t*_0_ + 8 s–*t*_0_ + 11 s) (stage 4–7 in Fig. 3c), finally forming a cavity in the SiO_2_ structure (Fig. 3a, *t*_0_ + 12 s) (stage 8 in Fig. 3c). In addition, the edge between the single and overlapped liquid Sn layer makes it easy to distinguish by contrast (Fig. 3a, *t*_0_ + 5 s–*t*_0_ + 8 s). Moreover, the as-formed cavity also shrunk with the further deformation of the SiO_2_ structure under electron beam irradiation (Fig. 3a, *t* = *t*_0_ + 26 s).

Under weaker electron beam irradiation (2.7 pA cm^−2^), another type of vanishing process of the Sn core was observed at 300 °C in a vacuum, named mode II, as shown in Fig. 3b, which is captured from the movie (ESI Movie 4, see the ESI†). Fig. S5a† shows that a Sn@SiO_2_ core–shell nanoparticle (core diameter: *ca.* 90 nm and shell thickness: *ca.* 8 nm) (blue arrow) with a small inner cavity and numerous hollow-structured SiO_2_ nanoparticles surrounding it was selected. The whole hollowing process is illustrated in Fig. 3d, in which Sn cores are outlined with orange dashed lines. In the beginning, a part of the liquid Sn core was already diffused out, forming a cavity beneath the SiO_2_ shell (Fig. 3b, *t* = *t*_0_) (stage 2 in Fig. 3d), and then the remaining Sn core decreased in size with time (Fig. 3b, *t*_0_ + 60 s–*t*_0_ + 479 s) (stage 2–4 in Fig. 3d) until the Sn core disappeared completely (Fig. S5b, see the ESI†) (stage 6 in Fig. 3d). In this process, the free surface of the Sn core, which was initially concave, turned nearly flat and finally became convex. According to the observation, Sn may diffuse out through the interconnected SiO_2_ shell structure (Fig. S5a, see the ESI†) since no leakage was observed and no Sn particles formed outside during our observation. Moreover, the wetting angle that is shown in Fig. 3b at *t* = *t*_0_ + 60 s was less than 90° and kept changing as heating proceeded, displaying dynamic wetting conditions on the curved inner surface of the shell. Combined with the results from Fig. 3a, this indicates that the embedded Sn droplets can completely wet the inner surface of the SiO_2_ shell in the as-prepared Sn@SiO_2_ core–shell structure.

This cavity formation realized by vanishing of Sn cores through simply raising the temperature could be a facile method to create a hollow structure. Several previous studies showed *in situ* observation of core-removal hollowing processes including using the Kirkendall effect,^14,15,24^ local etching of cores,^16^ evaporation of cores,^17^ draining away of liquid cores,^25^*etc*. Our observation showed a core-removal hollowing process by diffusion of liquid-state metal cores inside the shell structure, which has not been reported to the best of our knowledge.

**Fig. 4:**
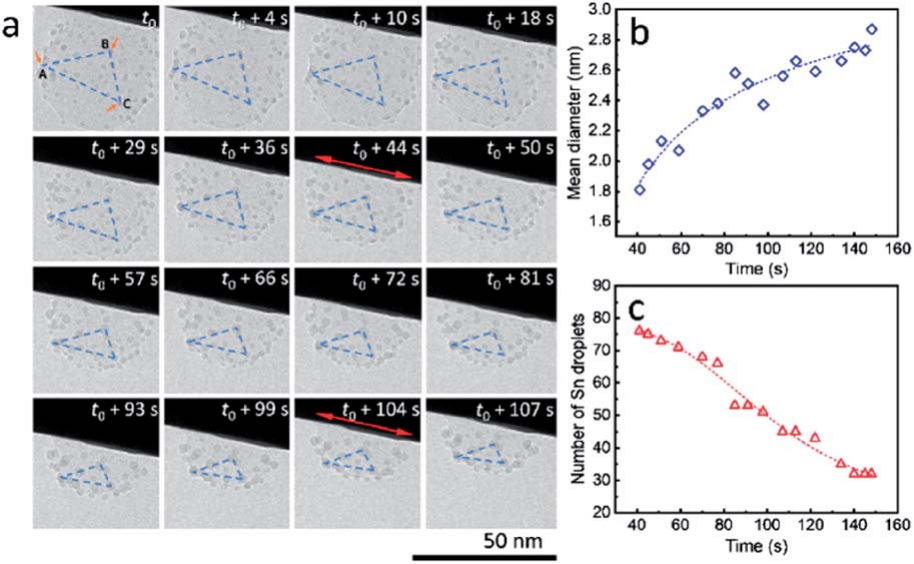
(a) Disappearance and growth of ultra-small Sn nanodroplets formed after disappearance of Sn cores during *in situ* TEM observation (strong electron beam conditions, 37.3 pA cm^−2^) at 300 °C in a vacuum. The change in shape and size of the blue dashed line triangle with vertices *A*, *B* and *C* located at the positions of three typical particles (*t* = *t*_0_) indicates the shrinkage of the SiO_2_ structure during the observation. (b) Increase in the mean diameter and (c) decrease in the number of ultra-small Sn nanodroplets in the observation field throughout the duration. The starting time *t*_0_ is set to 41 s in both (b and c). The fitting curves (dashed lines) in (b and c) show increasing and decreasing trends, respectively.

It is worth mentioning that, during the heating process, ultra-small liquid-state Sn nanodroplets (diameter < 5 nm) appeared and were distributed in the SiO_2_ structure accompanied by the disappearance of Sn cores (Fig. 3a). To collect more information on this phenomenon, a SiO_2_ sphere distributed with ultra-small nanodroplets and attached on a tungsten wire was observed during heating (ESI Movie 5, see the ESI†). The captured images of morphology evolution are shown in Fig. 4a. During the heating process, the SiO_2_ structure kept shrinking with shape transformation from a sphere to a spherical cap (*t*_0_–*t*_0_ + 107 s). Meanwhile, no change in the area of the contact interface (red double arrow, *t*_0_ + 4 s and *t*_0_ + 104 s) was observed. To show this shrinkage more clearly, a blue dashed line triangle was drawn with vertices *A*, *B* and *C* located at the positions of three characteristic Sn nanodroplets (*t* = *t*_0_). The change in size and shape of these triangles shows the shrinkage of SiO_2_, and the relative positions of these triangles indicate that the SiO_2_ structure did not move but clung to the tungsten wire during the observation. The mean diameter of distributed Sn nanodroplets inside SiO_2_ in each image is measured to show the growth of the as-formed nanodroplets (Fig. 4b), which increased from 1.8 nm to 2.9 nm. The corresponding size histograms are shown in Fig. S6.† Notably, during our observation, bigger Sn nanodroplets grew with time while small ones gradually disappeared, resulting in a decrease in the total number of Sn nanodroplets (Fig. 4c). This phenomenon indicates the occurrence of Ostwald ripening which is the result of the reduction in interface energy by the diffusion process inside the SiO_2_ structure.^26,27^

### Controllable nanostructure of Sn@SiO_2_

**Fig. 5:**
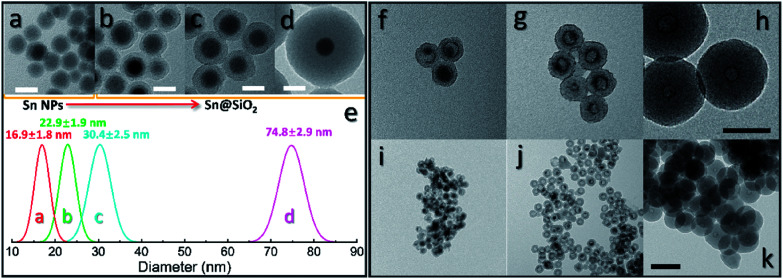
TEM images of (a) Sn nanoparticles used as the cores of Sn@SiO_2_ core–shell nanoparticles and Sn@SiO_2_ core–shell nanoparticles with shell thicknesses of (b) 3.0 nm, (c) 6.8 nm and (d) 29.0 nm, respectively. (e) Size histogram of Sn nanoparticles and Sn@SiO_2_ shown in (a–d). (f–k) TEM images of Sn@SiO_2_ core–shell nanoparticles, with various shell thicknesses ((f and i): 3.0 nm, (g and j): 6.8 nm, and (h and k): 29.0 nm), after loading in a sealed Al pan and heating from 25 °C to 300 °C at a ramping rate of 10 °C min^−1^ in a DSC. Scale bars for (a–d) are 20 nm, for (f–h) are 50 nm, and for (i–k) are 100 nm.

**Fig. 6:**
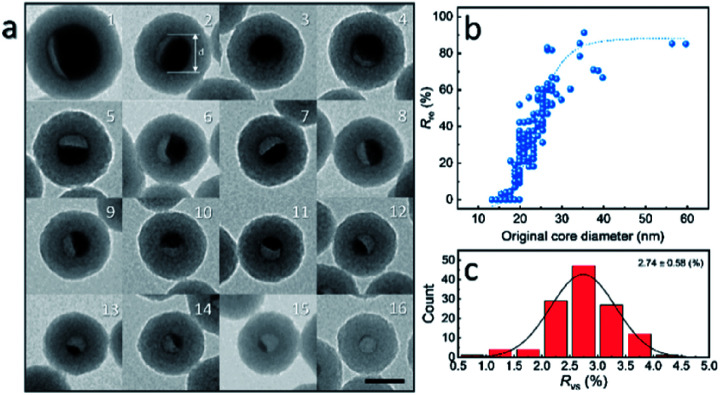
(a) After heating in a DSC (loaded in a sealed Al pan and heated from 25 °C to 300 °C at a ramping rate of 10 °C min^−1^), the Sn@SiO_2_ core–shell nanoparticles are transformed into yolk–shell (1–15) and hollow nanoparticles (16), showing an evolution from core–shell to yolk–shell and hollow nanoparticles. Sn@SiO_2_ core–shell nanoparticles with the same shell thickness (29.0 nm) but various core diameters (20–59 nm), shown in a-2 as “*d*”, are used as starting materials. The scale bar is 50 nm. (b) Volume ratio between the residual Sn core and the original Sn core (*R*_re_) obtained by measuring more than 200 particles with various diameters from 13.3 to 59.7 nm. The increasing ratio of the remaining Sn core with a bigger original core diameter indicates that Sn@SiO_2_ nanoparticles with bigger Sn cores will retain more Sn after heating, showing controllable Sn diffusion based on size difference. (c) Histogram of volume ratios between the as-formed void and shell (*R*_vs_) which is close to Gaussian distribution. The mean value of *R*_vs_ is 2.74 ± 0.58, indicating a possible Sn containing capacity of the SiO_2_ structure. The volume of the residual Sn core was calculated with the method given in the ESI.

To further investigate the formation of the hollow structure *via* Sn diffusion in the SiO_2_ structure, Sn@SiO_2_ core–shell nanoparticles with various shell thicknesses (3.0 nm, 6.8 nm, and 29.0 nm) are synthesized (Fig. 5b–d). Fig. 5a shows that the mean diameter of the original Sn nanoparticles used for the cores of Sn@SiO_2_ is 16.9 nm. Fig. 5e shows the narrow size distribution of Sn nanoparticles and Sn@SiO_2_ nanoparticles. To form the hollow SiO_2_ structure, these three Sn@SiO_2_ samples were transferred to a sealed aluminum pan and heated in a differential scanning calorimeter (DSC) under fixed heating conditions (from 25 °C to 300 °C, ramping rate: 10 °C min^−1^, hold for 30 min at 300 °C, naturally cool down to 25 °C). The corresponding DSC results of the heating experiments are found in Fig. S7 (see the ESI).† In all samples, the endothermic peaks located at around 232 °C indicate the melting of Sn cores embedded inside the SiO_2_ shell. The melting temperature is determined from the intersection point of the initial base line and auxiliary line drawn through the linear section of the peak slope as shown by the black dashed line in Fig. S7.†^28^ The melting temperatures of Sn cores embedded in the SiO_2_ shell with shell thicknesses of 3.0, 6.8 and 29.0 nm are 228.0, 228.5 and 228.5 °C, respectively, which are lower than the melting temperature of bulk Sn (231.9 °C) due to the size-dependent melting effect. According to the model provided by Lai *et al.*, the melting temperature of Sn nanoparticles with a diameter of 16.9 nm is 220.9 °C, which is lower than our measured value.^19^ It is believed that the SiO_2_ matrix environment may influence the surface atom mean-square displacement (msd) of the Sn core particle, thus affecting the melting temperature of Sn.^29^ The endothermic peaks located at 199.6, 191.6 and 169.4 °C were found during the initial heating of the as-prepared Sn@SiO_2_ core–shell nanoparticles shown in Fig. S7a,† which may come from the desorption of residual organics and water. In comparison, the DSC curves of Sn@SiO_2_ after the heating experiment only show the peaks from the melting of Sn cores (Fig. S7b†). After heating, Sn@SiO_2_ with a 3.0 nm SiO_2_ shell was transformed into the yolk–shell structure as shown in Fig. 5f and i, in which most of the Sn cores remain inside the SiO_2_ shell but decrease in size. With a thicker shell (6.8 nm), the Sn@SiO_2_ shown in Fig. 5g and j contains a much smaller Sn core after the heat treatment. A big proportion of the SiO_2_ structure became hollow, and part of it had small Sn cores retained in it. As for the Sn@SiO_2_ with the thickest SiO_2_ shell (29.0 nm), no Sn cores are found after heating, only the hollow SiO_2_ structure remains. Based on these results, it is found that a larger proportion of the metal Sn core in core–shell Sn@SiO_2_ nanoparticles with a thicker SiO_2_ shell moves out under the same heating conditions. One possible reason for this phenomenon seems to be that the as-prepared SiO_2_ shell has the capacity for fixing a certain amount of the Sn element which comes from the melted Sn cores by heating above the melting point of Sn. To collect more evidence for this hypothesis, Sn@SiO_2_ core–shell nanoparticles with various core sizes but uniform SiO_2_ shells with a thickness of 29.0 nm are synthesized. After the heating process under the same conditions, we found that the morphologies of the as-obtained nanoparticles show an evolution from the solid Sn@SiO_2_ core–shell structure to the yolk–shell structure and hollow structure (Fig. 6a). These three structures can be distinguished by using the volume ratio (*R*_re_) between the residual Sn core and the original Sn core: *R*_re_ = 1 for core–shell nanoparticles, 0 < *R*_re_ < 1 for yolk–shell nanoparticles and *R*_re_ = 0 for hollow nanoparticles. Fig. 6b shows that when the shell thickness is fixed, nanoparticles with higher *R*_re_ will be obtained with a bigger size of the original Sn core after the heating process. That is to say, Sn@SiO_2_ core–shell nanoparticles with relatively larger Sn cores may retain a greater proportion of the Sn core after the heating process. The volume of the residual Sn core was calculated by assuming it to be a spherical cap with the method given in the ESI.†^30^ Combined with the results from Fig. 5, we found that the amorphous SiO_2_ shell that was fabricated through the wet chemistry method in this case provides space with a certain capacity for fixing the Sn element after heating above the melting temperature to the liquid state. Silica that was made from wet chemistry methods generally contains physically adsorbed water molecules which are connected to the hydroxyl groups on the surface of the silica structure by hydrogen bonding, based on Zhuravlev's models.^31^ With this consideration, as discussed in Fig. 1f, SnO_2_ is observed after heating in a vacuum at 300 °C. This formation of SnO_2_ may be contributed by the interactions between liquid state Sn and the physically absorbed water molecules and hydroxyl groups on the surface of silica,^31–33^ as shown in the following equation:

1 Sn + 2H_2_O → SnO_2_ + H_2_↑

The as-formed SnO_2_ can easily get attached on the silica surface with hydroxyl groups. This phenomenon is different from that in our previous study, in which Sn nanoparticles were confined in porous silica spheres.^32^ In this case, the SiO_2_ matrix structure was pre-calcined, in which physically adsorbed water was removed, and after melt–freeze cycles of Sn nanoparticles, no morphology change occurred. During the heating process, as the physically adsorbed water is gradually consumed, the oxidation reaction of Sn tends to stop. Moreover, the as-formed SnO_2_ may fill and block the channels for the movement of liquid state Sn, thus keeping the rest of the Sn core unmoved so that the half-core or yolk–shell Sn@SiO_2_ structure can be formed. This yolk–shell structure formation indicates that the amount of Sn exceeds the Sn containing capacity of the shell, making the shell “saturated”. To quantify this capacity, the volume ratio (*R*_vs_) between the as-formed void (regarded as equal to the volume of moved-out Sn) and SiO_2_ shell after the heating process is used here, which is determined from the amount of the Sn element distributed in a unit volume of the SiO_2_ shell. Fig. 6c shows the distribution of *R*_vs_, which is calculated using the measured data from more than 200 Sn@SiO_2_ yolk–shell nanoparticles. The mean value of *R*_vs_ is calculated to be 2.74, based on which the maximum diameter of the original Sn core for the formation of the hollow structure with a SiO_2_ shell thickness of 29.0 nm is calculated to be 17.9 nm.

**Fig. 7:**
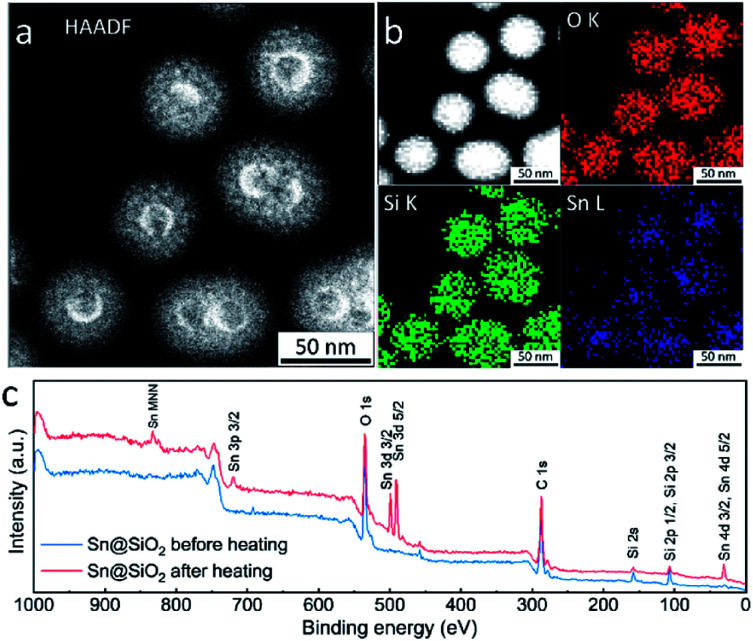
(a) HAADF-STEM image and (b) EDS element mapping images of the as-formed hollow structure obtained by ex situ TEM observation, showing the distribution of Sn, Si and O, respectively. The top left image in (b) is the map of superimposed signals from Sn, Si and O. (c) The XPS wide scan of Sn@SiO_2_ core–shell nanoparticles before and after heating.

High-angle annular dark-field scanning transmission electron microscopy (HAADF-STEM), energy dispersive spectroscopy (EDS) elemental mapping and X-ray photoelectron spectroscopy (XPS) were used for investigating the element distribution of the as-formed hollow SiO_2_ structure. The HAADF-STEM image in Fig. 7a shows a high *Z* (atomic number) contrast of the inner surface in the hollow SiO_2_ structures. Meanwhile, many bright spots in the shell area are found, indicating the existence of the embedded Sn element which has a higher *Z* contrast than SiO_2_ (Fig. 7a and S8d†). The elemental map in Fig. 7b shows that the Sn element is uniformly distributed inside the SiO_2_ hollow structure. During ex situ observation, similarly, the deformation of SiO_2_ is found: shrinkage occurred, and the surface became rough after electron beam focusing (Fig. S8a and b†). Fig. S8e† and the inset image show the HRTEM image and its corresponding fast Fourier transform (FFT) pattern, respectively. The interplanar distance is calculated to be 0.343 nm, corresponding to the (110) crystal planes of tetragonal SnO_2_ (JCPDS no. 41-1445) which is in good agreement with the SAED results shown in Fig. 1f and S4.† To check the Sn distribution on the surface and near surface area (3–10 nm), an XPS wide scan was performed on Sn@SiO_2_ core–shell nanoparticles before and after heat treatment in a DSC (Fig. 7c). Before heat treatment, no signal from Sn was detected, while a strong signal from Sn appeared when testing the Sn@SiO_2_ core–shell nanoparticles after heat treatment, showing strong evidence for outward diffusion of Sn. In addition, the XPS narrow scan (Fig. S9†) shows that most of the Sn in the hollow structure existed as SnO_2_,^34^ which is in good agreement with the previous discussion.

**Fig. 8:**
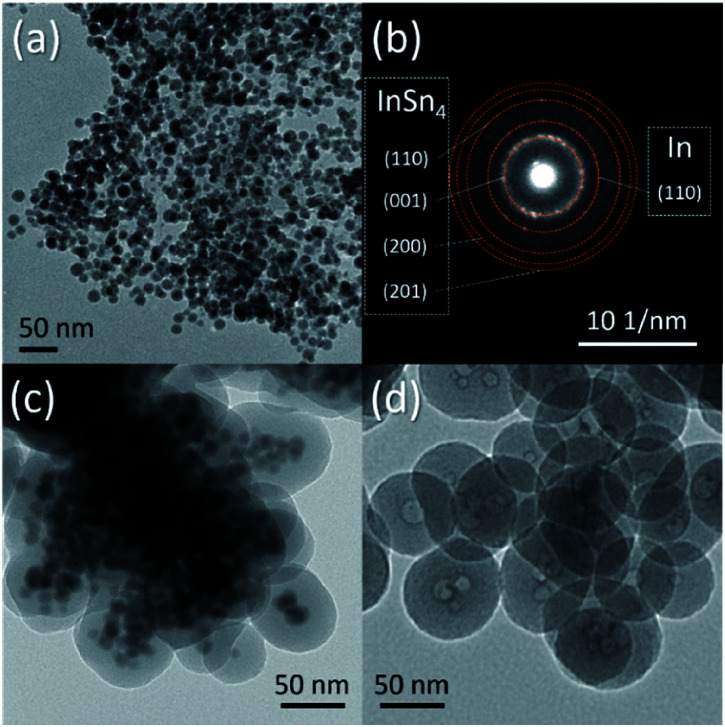
TEM image of the as-synthesized (a) In–Sn alloy nanoparticles and (c) In–Sn@SiO_2_ core–shell nanoparticles. (b) SAED pattern of the as-synthesized InSn_4_ nanoparticles. (d) InSn_4_ nanoparticles after heating in a DSC (from 25 °C to 300 °C, 10 °C min^−1^, loaded in a sealed Al pan).

It must also be mentioned that In–Sn@SiO_2_ (In–Sn alloy nanoparticle coated with SiO_2_) nanoparticles were prepared by a similar method (Fig. 8a–c). The In–Sn alloy has an even lower melting temperature. We applied the same conditions of the heating process as for Sn@SiO_2_ on In–Sn@SiO_2_. The hollow structure also appeared after being heated above the melting temperature (Fig. 8d), indicating that the diffusion of both In and Sn occurred inside the SiO_2_ structure, showing a potentially facile method for nanostructure control of low melting alloy (LMA) nanoparticles embedded inside SiO_2_ (LMA@SiO_2_).

## Conclusions

In summary, controllable nanostructure evolution of Sn@SiO_2_ from core–shell to yolk–shell and hollow has been achieved by the interaction between the liquid metal Sn and SiO_2_ shell structure synthesized through a wet chemistry method. The formation of a cavity inside the SiO_2_ structure and transformation of Sn@SiO_2_ core–shell nanoparticles into hollow SiO_2_ nanostructures by vanishing of liquid Sn cores inside Sn@SiO_2_ was observed in real-time *via in situ* TEM. The fixing of liquid state Sn inside the SiO_2_ shell structure takes the role of removing the cores in core–shell nanoparticles thus creating the cavity for the hollow nanostructure. Importantly, based on our findings, we demonstrated control over the morphologies of hollow/yolk–shell Sn@SiO_2_ nanostructures by adjusting the volume ratio between the core and shell of starting Sn@SiO_2_ core–shell nanoparticle precursors. Furthermore, our findings provide important knowledge of cavity formation through heating low melting point metal nanoparticles inside a SiO_2_ structure, as well as a potential tool for designing complicated hollow nanostructures in future studies.

## Experimental

### Synthesis of Sn nanoparticles

The Sn nanoparticles were synthesized using a well-developed method.^18^ 25 mL of oleylamine was vacuum dried in a 50 mL three-necked flask at 140 °C for 1.5 h and then naturally cooled down to 30 °C. Anhydrous SnCl_2_ (0.5 mmol) (min. 98%, Junsei) was added into the flask before heating to 140 °C and holding for an extra 30 minutes to remove the moisture. Afterwards, the flask was heated to 190 °C under an atmosphere of argon with constant stirring, followed by injection of 3.6 mmol lithium bis(trimethylsilyl)amide (LiN(SiMe_3_)_2_) (1.0 M in toluene, Aldrich) and a second injection of 0.6 mmol diisobutylaluminium hydride (DIBAH, 1.0 M in toluene, Kanto) within 10 s. The reaction solution became brown and then gradually turned black. The solution was stirred at 180 °C for 1 h to ensure the near-complete reaction of the metal precursor and then cooled down to 50 °C in an ice-water bath. 500 μL of dried oleic acid (OA, Junsei) were added before cooling down to 30 °C. The obtained solution was washed with ethanol and centrifuged (8000 rpm, 5 min). The precipitate was redispersed in a solvent mixture (6 mL, volume ratio: OA : hexane = 1 : 50) and then washed with ethanol. Finally, the purified precipitate was redispersed in 25 mL of hexane to form a stock solution of Sn nanoparticles. In addition, InSn_4_ nanoparticles were prepared in a similar way by using InCl_3_ mixed with SnCl_2_ in a molar ratio of 1 : 4 as the precursor.

### Synthesis of Sn@SiO_2_ core–shell nanoparticles

To encapsulate Sn nanoparticles with a uniform SiO_2_ shell, a modified reverse micro-emulsion method was used. A typical 12 mL Sn nanoparticle-dispersed stock solution was mixed with 0.61 g (0.594 mL) of polyethylene glycol mono-4-nonylphenyl ether (*n* = approx. 5, TCI, Tokyo, Japan) and 62 μL of deionized water (purified using a PureLabo system, Organo/ELGA, >18 MΩ). This emulsion was sonicated for 15 min to produce a good dispersion of the nanoparticles without aggregation. Afterward, 50 μL of TEOS (tetraethyl orthosilicate) were added under vigorous stirring (800 rpm). After 30 min hydrolysis and condensation of TEOS were triggered upon injection of 30 μL of an ammonia solution (min. 28.0%, Junsei, Tokyo, Japan). The mixture was stirred at 400 rpm for 10 h. Afterward, the obtained samples (Sn@SiO_2_ core–shell nanoparticles) were washed with ethanol three times and finally dried in a vacuum at room temperature. In particular, the thickness of the SiO_2_ shell can be easily tuned by changing the concentration of the Sn nanoparticle-dispersed stock solution.

### *In situ* TEM observation

*In situ* TEM observations were carried out using a JEM-1000K RS TEM (JEOL) operated at 1000 kV. A wire-type heating holder (JEOL, EM-Z081834SWHH) was used. The wire filament was heated using a DC generated by dry cells. The filament temperature was fixed by the calibrated current value.^35–37^ The powder samples (Sn@SiO_2_) were loaded on a tungsten wire and heated up to 300 °C under vacuum. TEM images were recorded with a charge-coupled device with an exposure time of typically 0.5 s and 5 s. The electron beam current flux for strong electron beam conditions was 37.3 pA cm^−2^, and that for weak electron beam conditions was 2.7 pA cm^−2^, as measured with a faraday gauge.

### *Ex situ* TEM observation

The *ex situ* TEM observation of Sn@SiO_2_ was carried out using a conventional TEM (JEM-2000FX microscope, JEOL) operated at 200 kV and a high-resolution field-emission gun scanning TEM (STEM, JEM-ARM200F Cold, JEOL) operated at 200 kV at room temperature and under a pressure of 10^−6^ Pa.

## Conflicts of interest

There are no conflicts to declare.

## Supplementary Material

NA-002-C9NA00782B-s001

NA-002-C9NA00782B-s002

NA-002-C9NA00782B-s003

NA-002-C9NA00782B-s004

NA-002-C9NA00782B-s005
